# Environmental Monitoring and Analysis of Faecal Contamination in an Urban Setting in the City of Bari (Apulia Region, Italy): Health and Hygiene Implications

**DOI:** 10.3390/ijerph7113972

**Published:** 2010-11-09

**Authors:** Elvira Tarsitano, Grazia Greco, Nicola Decaro, Francesco Nicassio, Maria Stella Lucente, Canio Buonavoglia, Maria Tempesta

**Affiliations:** Department of Veterinary Public Health, Faculty of Veterinary Medicine, University of Bari, Str. Prov. Casamassima Km 3, 70010, Valenzano, Bari, Italy; E-Mails: g.greco@veterinaria.uniba.it (G.G.); n.decaro@veterinaria.uniba.it (N.D.); nicassio@bleuline.it (F.N.); mariastella.lucente@libero.it (M.L.); c.buonavoglia@veterinaria.uniba.it (C.B.); m.tempesta@veterinaria.uniba.it (M.T.)

**Keywords:** public health, urban ecosystems, *Chlamydophila psittaci*, *Reovirus*, *Rotavirus*, *Toxocara canis*, *Toxascaris leonina*, *Ancylostoma caninum*, *Trichuris vulpis*

## Abstract

Few studies have been conducted in Italy to quantify the potential risk associated with dynamics and distribution of pathogens in urban settings. The aim of this study was to acquire data on the environmental faecal contamination in urban ecosystems, by assessing the presence of pathogens in public areas in the city of Bari (Apulia region, Italy). To determine the degree of environmental contamination, samples of dog faeces and bird guano were collected from different areas in the city of Bari (park green areas, playgrounds, public housing areas, parkways, and a school). A total of 152 canine faecal samples, in 54 pools, and two samples of pigeon guano from 66 monitored sites were examined. No samples were found in 12 areas spread over nine sites. *Chlamydophila psittaci* was detected in seven canine and two pigeon guano samples. Salmonella species were not found. On the other hand, four of 54 canine faecal samples were positive for reovirus. Thirteen canine faecal samples were positive for parasite eggs: 8/54 samples contained *Toxocara canis* and *Toxascaris leonina* eggs and 5/54 samples contained *Ancylostoma caninum* eggs. Our study showed that public areas are often contaminated by potentially zoonotic pathogens.

## 1. Introduction

The number of domestic animals in urban areas has progressively increased and dog faeces are not systematically removed from the streets, representing an important environmental pollution factor. An Eurispes survey revealed that 44,000,000 domestic animals, including 6,900,000 dogs, were living in Italy in 2002 (www.eurispes.it). The average daily faecal production of a dog is approximately 100 grams and open spaces, public gardens, pathways, arcades, bumps, pedestrian precincts and roadways are the sites with the highest pollution [1–3]. Canine faeces, not removed due to the bad habits of the owners, may represent a source of potential pathogens in addition to an inconvenience to people [1,4–8]. Viable pathogens in dried and pulverized canine faeces can be spread by wind, vehicular traffic and can be a source of infection through unwrapped food. They also can be carried inside houses via contaminated shoes [1,4]. Children, the elderly and immunocompromised people are at greatest risk.

Another important aspect concerns the colonization of cities by synanthropic species such as rodents and pigeons. In urban cities, several favourable environmental situations occur that lead to a real demographic explosion of animal populations and related borne infections [9]. The multiplicity of ecological niches in an urban environment encourages the increase of certain offensive animal species as a consequence of more optimal temperatures for reproduction and growth, as well as the abundance of food and water. This situation promotes overcrowding and the increase in individuals of the same species so that they create a problem by inducing the emergence and spreading of new pathogens and allergies [10–12]. Synanthropic animals such as pigeons (*Columbia livia)*, have increased their number so highly that specific control measures have been requested by the city of Bari, and elsewhere [13–17]. Their adaptation to the urban habitat and colonization of new ecological niches has lead to the accumulation of a large quantity of guano in the plazas, on monuments and buildings and in every place used for nest building. It is well known that guano can represent a biological and health risk for humans and animals since it may be a source of viral, bacterial and fungal infections, as well as a substrate for ectoparasites [11–13,18,19].

Free-living pigeons are potential reservoirs for several pathogenic microorganisms, including *Chlamydophila psittaci* and bacteria belonging to the genus *Salmonella*. In Japan, *S. typhimurium* and *C. psittaci* have been isolated with a high frequency from feral pigeons [20,21]. Recent studies performed in feral pigeons in Italy, Bosnia, Herzegovina, and Macedonia showed a seropositivity of 48.5%, 26.5% and 19.2%, respectively [22–24]. *C. psittaci* DNA also has been detected in the faeces (16%) of feral pigeons in north-eastern Italian towns [25]. Until now, no systematic investigations have been reported on pathogenic agents in faeces shed from pigeons in south-eastern regions of Italy. During 2006–2007, we conducted extensive surveys for pathogens, especially *Salmonella* and *Chlamydia*, in pigeon faeces collected from public areas in Bari, Italy. Canine faecal samples were included in the survey because of the high presence of dog faeces in the urban setting.

Mammalian orthoreoviruses (MRV) have been sporadically reported in different countries, including Italy. MRV were included in the present study since they have a broad host range and wide geographic distribution [26,27]. The overall aim of the present study was to obtain information on urban hygiene and ecology, the extent of environmental faecalization, the potential spread of transmissible diseases from those animals to humans and other animal species and the zoonotic risk associated with animals living in the urban area. Data arising from the study could be useful to evaluate the environmental and public health hazards and identify specific control measures against these diseases offering management and control measures in an integrated and participatory way in order to launch public awareness-raising campaigns [9].

## 2. Materials and Methods

The survey was planned in several steps that included territorial mapping, monitoring and sampling and analytical surveys.

### 2.1. Territorial Mapping

**Figure 1:**
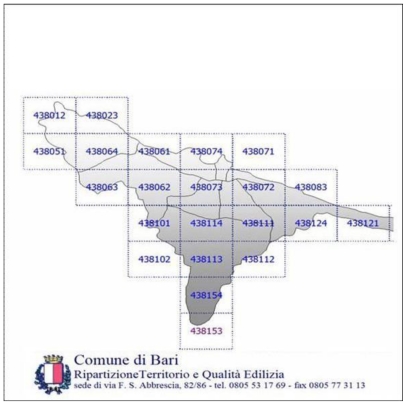
Map of the study area showing the sampling sites.

An initial analysis of the urban Bari was conducted by using an appropriate cartography (Municipality of Bari) utilizing the cadastral maps (1:5,000) of the city (see Figure 1).

**Figure 2:**
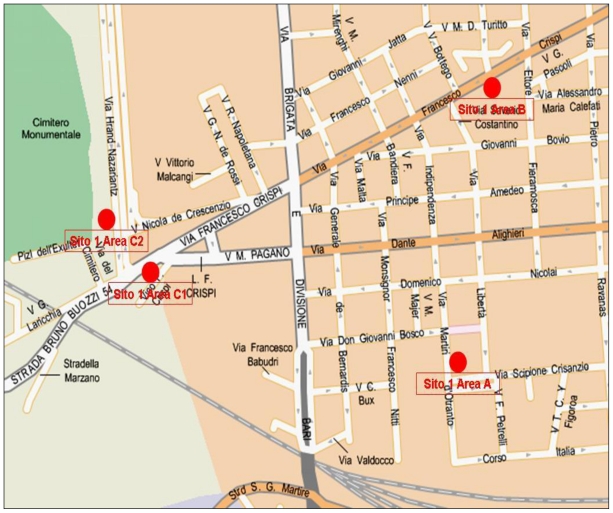
An example of street map of one site, areas A, B, C. Red points represent the canine faecal collection points.

The final choice took into account the field evaluation of the areas. A preliminary division of the territory into 22 sites (numbered from 1 to 22) was made, corresponding to the different districts of Bari, and based on the available cartography. Each site was subdivided into three areas (A, B and C), taking into account the environmental characteristics of each district: a very densely populated area with poor green; an intermediate anthropized green area; a highly decay area (see Figure 2).

In order to evaluate the possibility of transmission of zoonotic infections to the most susceptible people, areas used for play by children were chosen for monitoring. Each area was fixed for vegetation, size (square meters), presence of restricted areas for dogs, frequency of cleaning and collection of dog excreta by the municipality. On this basis, it was possible to plan the number of faecal samples to be collected and analysed per area. The division of the municipal land into sites and areas was carried out directly and with the help of Bari cartography, between the 15th of April and the 15th of November. Furthermore, global positioning system (GPS) grid references were calculated for each area to expedite further mapping, monitoring and sampling (see Table 1).

### 2.2. Monitoring and Sampling

First, sampling areas were chosen, mapped and divided. Then, sampling was carried out for the detection of pathogens (reoviruses and rotaviruses, chlamydial agents, *Salmonella* spp. and parasites) in the guano of birds (*Columbia livia*) and/or canine faeces. A pool of one to five samples of dog faeces (with an average of three) collected in each area was processed for laboratory analysis. No faecal specimens were found in 12 areas that were spread over nine sites. A total of 152 canine faecal specimens were collected and pooled into 54 samples. Only three sites were identified where there were stable and populated colonies of pigeons from which guano samples were collected for analysis.

### 2.3. Laboratory Analyses

All analyses were carried out in the laboratories of the Section of Infectious Diseases and Parasitic Diseases of the Department of Veterinary Public Health of the Faculty of Veterinary Medicine of the University of Bari. The 54 canine faecal samples and the three pigeon guano samples (S1.C2, S2.B e S5.B) were processed for bacteriological, virological and parasitological examinations.

#### 2.3.1. DNA Extraction

For analysis, total DNA was extracted from the faecal and guano samples using the QIAmp Tissue kit (QIAGEN GmbH, Germany) according the manufacturer’s instructions. All PCR assays were performed in a DNA Thermal Cycler Gene Amp 9600 (Perkin Elmer Cetus, Norwalk, CT, USA). PCR products were electrophoresed on a 2% agarose gel, stained with ethidium bromide and visualized in a Gel Chemidoc analysis system (Bio-rad srl, Milan, Italy).

#### 2.3.2. Bacteriological Investigations

Dog and pigeon faecal samples were analyzed for *Chlamydophila* spp and *Salmonella* spp. DNAs by a polymerase chain reaction (PCR). Detection of *Chlamydophila* spp was done by PCR, based on the *pmp*, able to detect *C. abortus*, *C. psittaci* and *C. caviae*, according to the method of Laroucau *et al.*, [28] using the pair of oligonucleotide primers CpsiA (5′-ATG AAA CAT CCA GTC TAC TGG-3) and CpsiB (5′-TTG TGT AGT ATT ATT ATC AAA-3′). RFLP analysis, able to obtain the characterization at species level, was performed on the PCR products of the reference strain *C. abortus* [29] and on the amplicons obtained from two samples of guano and seven canine faecal samples. For the detection of *Salmonella* spp., a PCR assay specific for the *inv*A gene of *Salmonella* spp. was used [30].

#### 2.3.3. Virological Investigations

Reovirus RNA was extracted using the guanidine thiocyanate/Glass Milk method, following the protocol described by Gentsch *et al*. [31]. For the detection of RV a nested-PCR assay was performed [32], using primer pairs L1-rv5/L1-rv6 for RT-PCR and L1-rv7/L1-rv8 for nested-PCR. The detection of rotaviruses was performed as previously described [33].

#### 2.3.4. Parasitological Investigations

Sample analysis was performed using a zinc chloride solution (d = 1,350). Furthermore, a quantitative coprologic analysis of the positive samples was carried out by means of the Mc Master method [34].

## 3. Results and Discussion

Pathogens were detected in 23 out of 66 monitored urban areas (34.85%), showing that organisms potentially hazardous for public health indeed contaminate public areas.

### 3.1. Bacteriological Investigations

Out of all the samples collected in the studied areas, only two pigeon guano (S1.C2 e S5.B) and seven canine faecal samples (S1.B, S1.C1, S2.C, S3.B, S20.C, S21.A e S22.A) (see Table 1) were positive for *Chlamydophila psittaci* (12.96%). *Salmonella* spp. were never found in any of the samples analysed.

### 3.2. Virological Investigations

**Figure 3:**
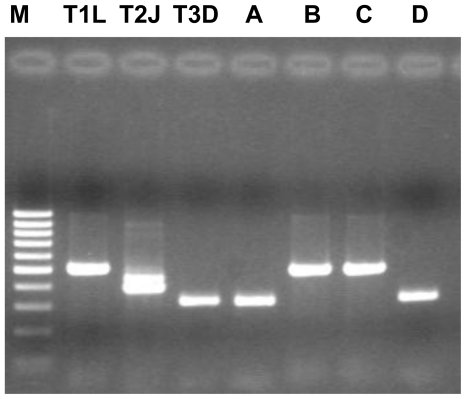
Reovirus strains characterization by the type 3-specific RT-PCR assay targeting the S1 segment (primer pair S1-R3F/S1-R3R, PCR product of 326 bp).

Two guano samples were negative for reovirus and rotavirus. Four canine samples (S8.A, S12.A, S17.B e S22.A) were positive for mammalian reoviruses (7.40%) (see Table 1, Figure 3). No rotavirus strains were found in any faecal sample.

### 3.3. Parasitological Investigations

**Figure 4:**
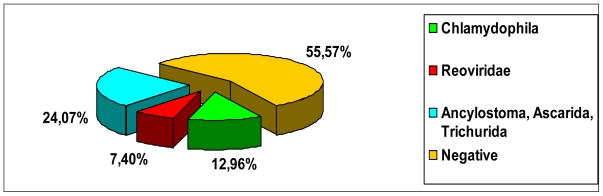
Percentages of the different pathogens isolated from canine faeces and bird guano.

Thirteen out of 54 samples were positive for parasites (24.07%). Specifically identified were: Ascarid eggs (*Toxocara canis* and *Toxascaris leonina*), found in eight of 54 samples and hookworm eggs (*Ancylostoma caninum*) identified in five samples. Three samples were positive for canine whipworms (*Trichuris vulpis*) that are not hazardous for man; cestode eggs were not found in any case. A canine faecal sample resulted positive for coccidial oocysts (genus *Isospora)* that are not considered important for public health. The concurrent presence of two different parasite species was observed (mixed infestations) in three of 54 samples; in two samples, ascarida and ancyilostoma and in another sample ascarida and trichurida were found. All guano samples were negative for parasites. Overall results are reported in Table 2 and Figure 4.

## 4. Conclusions

We have demonstrated, by PCR analysis of bacterial DNA in faecal samples, that animals within the city of Bari may be carriers of *Chlamydophila*. Family *Chlamydiaceae* includes nine species, some of which are responsible for human diseases; others are pathogenic only for animals.

Interspecies circulation has been clearly documented for chlamydial agents and they may cause severe disease when transmitted to different animal species [35,36]. For example, pigeons (*Columba livia*) infected by *Chlamydophila psittaci* seldom show clinical signs, but often are able to spread the organisms in the environment, enhancing the risk of transmission to humans [35,36]. The presence of *Chlamydophila* spp. in canine faeces raises some questions since it is unknown how dogs can be infected by these pathogens. Hypothetically, dogs could be infected because of their behaviour of sniffing and licking everything within reach, including sources potentially contaminated by infected pigeon guano. It is not possible to rule out contamination of canine faeces by dried pigeon guano that had been spread in the air.

In this study, it was not possible to establish a link between the presence of *C. psittaci* in the faeces and the health status of carrier animals. In a manner similar to dogs that share urban areas with birds, it is possible that humans may come in contact with pathogens shed by dogs, especially where they live together in close quarters. Human infections by *C. psittaci,* been reported on several occasions, especially in people who handled birds. Nevertheless, additional study is needed in order to clarify the pathogenic role of *C. psittaci* strains for dogs and, more importantly, to assess the zoonotic risk for humans. Furthermore, considering that microorganisms carried by asymptomatic dogs and/or birds could infect humans, an intensification of surveillance on their circulation in urban animals is needed. It should be stressed that human demographic and behavioural changes may favour the emergence and spread of new pathogens [37].

Reoviruses are ubiquitous and poorly species-specific viruses [38]. Up to now, three distinct serotypes, T1, T2 e T3, have been identified in mammals. Although such viruses could be isolated either from respiratory and enteric tracts, a specific disease has been not yet associated with reovirus infection. Reovirus infections of the respiratory and enteric tracts are generally asymptomatic or mild. The potential association of reoviruses with extra-hepatic biliary atresia and, especially, with neurologic and exanthematic forms must be still further evaluated. In humans, both T1 and T3 strains have been associated with neurological forms and only recently, a T3 strain has been isolated from a child suffering from a severe meningoencephalitis. The positivity of these samples shown only by nested PCR demonstrates the presence of a low viral titre in the canine faeces and, likely, subclinical infections of the dogs. Even though the pathogenicity and the zoonotic potential of reoviruses are still unknown, the detection of reoviruses in faecal samples of dogs cannot be minimized with regard to the possible implication for public health. It is well known that several factors, such as human contact with the pathogen and the genetic adaptation to the human host, play an important role in the process of viral zoonoses, providing (within mechanisms of interspecies transmission) favourable conditions to a pathogen for emerging [39].

The results of parasitologic analysis showed the presence of *Toxocara canis* and *Toxascaris leonina*, which are potentially responsible for human illness (*larva migrans* syndromes). In fact, they can be transmitted from dogs to humans, giving even severe infestations. *Toxocara canis* and *Toxascaris leonina* cause visceral *larva migrans* [40,41]. The ingestion of embryonated eggs by humans results in somatic migration of larvae that can remain dormant in several tissues for long periods of time. In humans, the infection is usually asymptomatic but rare cases may be characterized as visceral *larva migrans* with fever, hepatomegaly, coughing and eosinophilia, or as ocular *larva migrans*, characterized by endophthalmitis and, sometimes, the formation of a retinal granulomatous mass. In addition to these classical syndromes, clinical signs may occur, including abdominal pain, anorexia, nausea, vomiting, sleep disturbance and behavioural distress, coughing, dyspnoea, pharyngitis, migraine and cervical adenitis [41–43]. *Ancylostoma caninum* can cause a form of dermatitis (“creeping eruption”). Several days following infestation, larvae migrate, creating an erythematosus-papular-vesicular serpiginous line which proceeds a few millimeters per day. Clinical signs are characterized by itching, often so intense that affected individuals cannot sleep and scratching may result in extensive papular-pustular dermatitis. Legs are usually the most affected areas due to high exposure to larval penetration [41,44].

The presence of large amounts of dog faeces Italian cities represents a major environmental problem from both hygienic and social points of views [4–7,45–47]. The level of pathogenic agents in the faeces of dogs and pigeon guano can be considered as an indicator of hazardousness for public health [7,8,42,45,48–52]. Results from this survey indicate the need to investigate the problems in the cities associated with human diseases that might be associated ecologically with synanthropic or domestic pets [9]. It is well known that unsustainable management of the cities, climatic changes, and intense urbanization can modify the biology of the hosts, pathogens and vectors increasing the transmission rate and the adaptation of the hosts to the urban environment with no marginal consequences [53]. Proper regional planning, which is part and parcel of an overall integrated management system of an urban environment, requires a thorough grasp of issues such as environmental faecal contamination in urban ecosystems which, in this particular case, becomes a factor impacting ecology, ethology and pathology [9]. It follows that all these issues must be studied from a number of viewpoints, in a multidisciplinary fashion. If current city-planning practice is to be sustainable, then it must involve and include the cooperation of experts from a broad range of fields: biologists, veterinarians, physicians, naturalists, city planners, economists and administrators [54,55]. Such assessments pertain, especially, to the levels of vulnerability, to the level of critical state and to the potential of the urban ecosystem under study [56]. At the end of the assessment stage, a sustainability matrix should be established (cause/effect and the incidence levels); intervention plans shall be drafted, including proposals for changing the current technical management levels of the interaction between the environment and animals in urban settings [57–59]. These are all considerations to be assessed when taking an ecosystemic approach to city planning [9]. A specific set of data should be used as a basis for the establishment of suitable hygiene and health criteria; on that basis, appropriate ‘safety factors’ should be built in, allowing for an extrapolation of test values to levels which can be safely considered protection safety levels for the environment and for public health [57,60,61]. These areas of expertise coming from various scientific disciplines must be coordinated and combined in order to ensure that appropriate planning strategies are drawn up to implement preventive measures and control measures for the best possible use of the urban environment. The same expertise can also be used as guidance tools to adjust sustainable city planning policies to foster quality of life, public health and the health of the environment [9].

## Figures and Tables

**Table 1:**
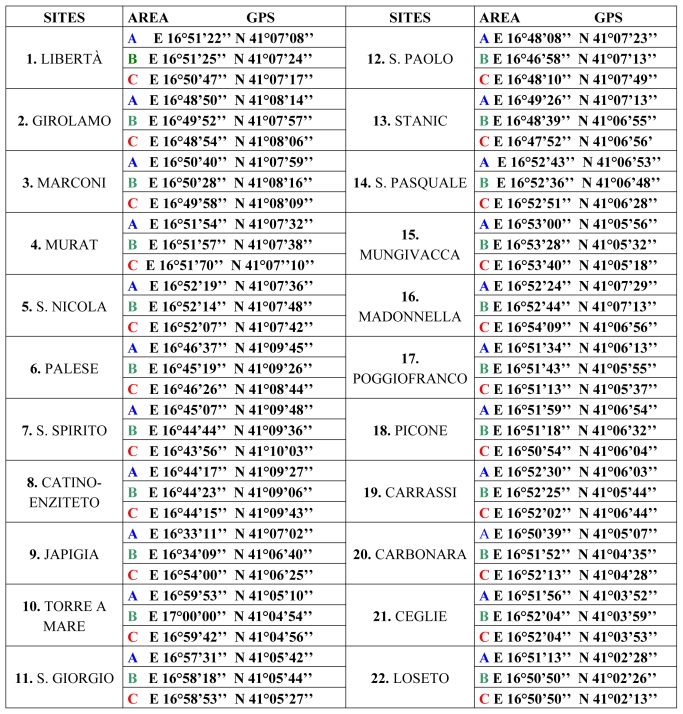
Cartographic district allocation in areas and sites in Bari. Twenty-two sites corresponding to different districts numbered 1 to 22. Each site was subdivided into three areas (A, B, C) for a total number of 66 areas, corresponding to streets, squares and avenues of the district. GPS coordinates were measured for the exact localization.

**Table 2:** Sites and areas, GPS position and pathogens isolated from canine faeces and bird guano samples analyzed.

SITES	AREA - GPS POSITION	ISOLATED PATHOGENS
**1. LIBERTÀ**	**B -** E 16°51′25″, N 41°07′24″	***C. psittaci***
	**C1 -** E 16°50′52″, N 41°07′13″	***C. psittaci***
	**C2 -** E 16°50′47″, N 41°07′17″	***C. psittaci*(Guano)**
**2. SAN GIROLAMO**	**B -** E 16°49′52″, N 41°07′57″	**Ancylostoma**
	**C -** E 16°48′54″, N 41°08′06″	***C. psittaci***
**3. MARCONI**	**B -** E 16°50′28″, N 41°08′16″	**Ascarida*C. psittaci***
**4. MURAT**	**A -** E 16°51′54″, N 41°07′32″	**Ascarida Ancylostoma**
	**C -** E 16°52′02″, N 41°07′13″	**Ancylostoma**
**5. SAN NICOLA**	**B -** E 16°52′14″, N 41°07′48″	**Ascarida** ***C. psittaci*(Guano)**
**6. PALESE**	**B –**E 16°45′19″ N 41°09′26″	**Ascarida**
**8. CATINO - ENZITETO**	**A -** E 16°44′17″, N 41°09′27″	**Reoviridae**
	**B -** E 16°44′23″, N 41°09′06″	**Ascarida Trichurida**
**10. TORRE A MARE**	**C -** E 16°59′42″, N 41°04′56″	**Trichurida**
**12. SAN PAOLO**	**A** - E 16°48′08″, N 41°07′23″	**Reoviridae Coccidia**
**17. POGGIOFRANCO**	**A -** E 16°51′34″, N 41°06′13″	**Ascarida Ancylostoma**
	**B -** E 16°51′43″, N 41°05′55″	**Reoviridae**
**18. PICONE**	**B -** E 16°51′18″, N 41°06′32″	**Ascarida**
**19. CARRASSI**	**A -** E 16°52′30″, N 41°06′03″	**Ascarida**
	**C -** E 16°52′02″, N 41°06′44″	**Ascarida**
**20. CARBONARA**	**C -** E 16°52′13″, N 41°04′28″	***C. psittaci***
**21. CEGLIE**	**A -** E 16°51′56″, N 41°03′52″	***C. psittaci***
	**B -** E 16°52′04″, N 41°03′59″	**Ancylostoma**
**22. LOSETO**	**A -** E 16°51′13″, N 41°02′28″	***C. psittaci*** **Reoviridae**
